# The application of the RE-AIM and PRISM framework to process evaluations of diabetes self-management programs: a systematic review and secondary analysis of literature

**DOI:** 10.3389/fpubh.2025.1588457

**Published:** 2025-12-12

**Authors:** Chinelo Nsobundu, Yeka Nmadu, Nikita Sandeep Wagle, Oluwafemi Aremu, Allen Eruaga, Ngalula Dolly Mwintshi, Mariam Olamide Salis, Margaret J. Foster, Matthew Lee Smith, Marcia G. Ory

**Affiliations:** 1 Center for Community Health and Aging, Texas A&M University, College Station, TX, United States; 2 School of Medicine, St. George’s University, St. George's, Grenada; 3 Department of Pediatrics, College of Medicine- Jacksonville, University of Florida, Jacksonville, FL, United States; 4 School of Public Health, Texas A&M University, College Station, TX, United States; 5 Ascension Wisconsin Family Health Center, Milwaukee, WI, United States; 6 Center for Systematic Reviews and Research Syntheses at the Medical Sciences Library, Texas A&M University, College Station, TX, United States

**Keywords:** diabetes self management, process evaluation, implementation, REAIM, PRISM

## Abstract

**Background:**

Diabetes self-management programs, often known as DSMPs, are crucial interventions for enhancing clinical and behavioral outcomes in individuals with diabetes. Despite the well-established advantages of these programs, little is known about how they are carried out and maintained in real world environments. This systematic review builds upon a previously published prequel by applying the RE-AIM (Reach, Effectiveness, Adoption, Implementation, and Maintenance) and PRISM (Practical, Robust Implementation and Sustainability Model) frameworks to examine the process evaluations of traditional, group-based DSMPs, with the goal of identifying implementation strengths, gaps, and contextual influences.

**Methods:**

Using five databases, a comprehensive analysis of peer-reviewed literature up to January 2024 was performed. Eligible studies included traditional, group-based DSMPs for adults with type 1 or type 2 diabetes that reported at least one process evaluation outcome. Sixty-eight studies (*n* = 78 articles) met inclusion criteria. Data were extracted and synthesized using RE-AIM and PRISM components, and study quality was appraised using the Mixed Methods Appraisal Tool (MMAT).

**Results:**

Sixty-eight studies (k = 68; *n* = 78) satisfied the requirements for inclusion. Fewer research addressed Adoption (k = 5), Implementation (k = 24), and Maintenance (k = 26), whereas the majority focused on Reach (k = 66) and Effectiveness (k = 66). Only four studies found external contextual influences, compared to nineteen that found internal contextual elements. Process-level indicators—such as implementation fidelity, cost, sustainability, and provider engagement—were often underreported, despite consistent evidence of positive clinical and behavioral outcomes.

**Discussion:**

The review highlights a critical gap between outcome evaluation and implementation reporting in DSMP research. The organizational and contextual elements that affect program scalability and durability were overlooked in favor of patient-level outcomes in most of the studies. When the RE-AIM and PRISM frameworks were used systematically, it was discovered that several process components that are essential for long-term integration were underreported. Resolving these shortcomings can help direct the creation of scalable, context-aware DSMPs that are more adaptable to diverse populations and settings.

**Conclusion:**

Process evaluations should be thorough, context-sensitive, and theory-driven to improve the implementation, execution, and long-term effects of DSMPs. A more comprehensive analysis of program success is made possible by integrating the RE-AIM and PRISM frameworks, which closes the gap between research and real-world implementation. To educate healthcare policy and practice, future evaluations should use mixed method designs that evaluate implementation as well as results.

**Systematic review registration:**

This systematic review was registered in PROSPERO. The registration number is CRD42020177170.

## 1 Introduction

Diabetes has become incredibly common, which presents a significant public health concern. Approximately 589 million adults aged 20–79 worldwide are projected to have diabetes (1). The hallmark of diabetes mellitus, a long-term metabolic disease, is hyperglycemia brought on by deficiencies in either insulin action or secretion, or both. Usually identified in childhood or adolescence, type 1 diabetes mellitus (T1DM) is an autoimmune disease in which the body’s immune system kills beta cells in the pancreas, resulting in complete insulin insufficiency. On the other hand, most cases are Type 2 diabetes mellitus (T2DM), which is more common in adults but is becoming more prevalent in young people. T2DM is principally linked to insulin resistance and progressive beta-cell dysfunction.

Diabetes Self-Management Programs (DSMPs) have emerged as pivotal interventions to empower individuals in navigating the intricacies of diabetes management (2–8). DSMPs can be broadly classified into two categories: diabetes self-management support (DSMS) programs and diabetes self-management education (DSME) programs. They are referred to as diabetes self-management education and/or support programs (DSMES) when used together. According to Powers et al. (9), DSME is the continuous process of facilitating the information, skill, and ability required for diabetes self-care, while DSMS helps the individual with diabetes adopt and maintain the behaviors required to continuously manage their condition.

Various studies emphasize that effective implementation of DSMPs is intricately linked with favorable outcomes. Improved hemoglobin A1C levels, enhanced health indicators, heightened self-efficacy, and the cultivation of proactive self-care behaviors are among the positive outcomes consistently associated with successful DSMP implementation (2–5, 10). This association underscores the potential of these programs to impact the health and well-being of individuals grappling with diabetes. However, it’s crucial to recognize that DSMP initiatives do not always lead to better results, though. Inconsistent outcomes may arise from variations in program fidelity, delivery strategies, participant involvement, and contextual elements, especially in real-world contexts. In this publication, we shall refer to all diabetes self-management-related programs as DSMPs because not all DSMES programs are accredited.

However, the positive results displayed from DSMPs are contingent upon the meticulous assessment of program implementation. In fact, successful DSMPs are not merely characterized by their existence but their fidelity and precision with which they are implemented. Therefore, process evaluations are essential as they provide a structured and methodical means to scrutinize the intricate dynamics of program implementation (11).

Although process evaluations in health-related interventions have been the subject of previous systematic reviews (12–17), less focus has been placed on the details of each process evaluation outcome in DSMPs, particularly those that use traditional, group-based techniques. Consequently, this systematic review aims to address critical research questions, exploring the knowledge gaps in stand-alone or embedded process evaluations, common process outcomes, and the methods employed in studies focused on traditional, group-based DSMPs. This review is in complete accordance with the methodical approach as it makes use of the PRISM Framework to disentangle the complexities of diabetes self-management treatments and advance knowledge on their efficacy and use. Evaluating DSMPs represents a critical endeavor in confronting the burgeoning global challenge of diabetes mellitus. Amidst various evaluation frameworks, the Practical, Robust, Implementation, and Sustainability Model (PRISM) offers a comprehensive approach to evaluating the implementation of DSMPs by considering the multilevel and dynamic interactions between an intervention, the perspective and characteristics of diverse recipients, the implementation and sustainability infrastructure, and the external environment (18, 19). In other words, PRISM provides a practical, actionable model used to plan and guide interventions, implementation strategies, adaptations, and factors related to sustainability (18, 19). Serving as an extension of the more widely known RE-AIM Framework, PRISM proposes that key contextual factors influence the RE-AIM outcomes (18, 19). Thus, these frameworks offers a nuanced lens to understand not only the immediate effectiveness of DSMPs but also their broader applicability, adoption, implementation fidelity, and long-term sustainability (18, 19).

For these reasons, a systematic review was conducted to synthesize, report, and apply the PRISM components to process evaluations of traditional, group-based diabetes self-management programs. In other words, the review explores (1) which process outcomes are commonly characterized in traditional, group-based DSMPs (Reach, Effectiveness, Adoption, Implementation, Maintenance, Internal and External Contextual factors) and to what dimension? and (2) What are the characteristics of those studies who utilize all versus some of the component dimensions?

This manuscript stems from a foundation laid by a prequel paper which summarized the essential elements of conventional, group-based diabetes self-management programs (DSMPs), such as program structure, target populations, delivery setting, interventionists, and curriculum components (20). Using a fundamental implementation science lens created by Saunders and colleagues, the prequel paper concentrated on broadly identifying and classifying process evaluation components—such as fidelity, dose delivered, dose received, and participant satisfaction—within traditional, group-based diabetes self-management programs (DSMPs) using the Implementation Monitoring & Process Evaluation guide (21, 22).

On the other hand, the current manuscript takes a more rigorous and granular approach by methodically utilizing the RE-AIM and PRISM frameworks to assess the degree to which these DSMPs incorporated key implementation and contextual factors. This paper critically evaluates the extent to which each study covered key areas including reach, effectiveness, adoption, implementation fidelity, maintenance, and internal and external context, rather than merely listing program elements. This offers a multifaceted overview of how real-world elements essential to translation, scale-up, and sustainability have been considered in DSMP evaluations.

Additionally, quantitative synthesis is introduced in this publication, which counts the number of RE-AIM/PRISM elements reported in each study and illustrates research diversity (e.g., how many studies reported 2 versus 6 elements). The previous paper lacked this degree of detail. The current paper also introduces implementation-focused implications and identifies gaps in contextual reporting, which are essential for informing future DSMP design and evaluation strategies.

## 2 Methods

This systematic review adhered to the Preferred Reporting Items for Systematic Reviews and Meta-Analyses (PRISMA) statement, ensuring transparency and rigor in the review process (20). The review protocol is registered *a priori* and is publicly available on the International Prospective Register of Systematic Reviews (PROSPERO) database (PROSPERO registration number CRD42020177170) (20).

### 2.1 Data sources

A comprehensive search strategy was developed by a Texas A&M University librarian with expertise in systematic reviews encompassing three concepts: diabetes, self-care, and implementation across various bibliographic databases from inception to January, 12, 2024. The databases included Medline (Ovid), Embase (Ovid), CINAHL (Ebsco), Academic Search (Ebsco), and APA PsycInfo (Ebsco). Each of these concepts were searched with multiple synonyms and keywords as appropriate for each database. The electronic search was supplemented by searching for relevant studies and reviewing grey literature. The search strategies for each database are published in a previously published systematic review and are in Appendix B of the Supplementary File (20).

### 2.2 Inclusion and exclusion criteria

Empirical studies were included based on specific criteria: (1) involvement of one or more traditional, group-based Diabetes Self-Management Programs (DSMPs), such as diabetes education programs, group medical visits, peer support groups, and hybrid models; (2) participation of adults aged at least 18 years with either T1DM or T2DM; (3) incorporation of a stand-alone or embedded process evaluation including at least one process outcome (e.g., fidelity, dose delivered, dose received, reach, retention, recruitment, and context); (4) employment of a quantitative, qualitative, or mixed-method study design; and (5) publication in the English language. The inclusion of adults aged 18 years and older aimed to diversify study retrieval and capitalize on their ability to self-direct learning, draw on life experiences, and apply new knowledge to real-life situations (23).

### 2.3 Study selection

After the same three researchers (including the main researcher) from the prequal paper screened the title and abstracts (20), those retained were subjected to full-text review. Forward and backward citations and additional searching via the grey literature were performed. Disagreements were resolved on study selection and extraction by consensus if needed.

### 2.4 Data extraction and quality appraisal

The main researcher created a thorough data extraction form, in which four researchers different from those who helped screen articles pilot-tested the extraction form before being used (24). Then, a total of five researchers (four different researchers & the main researcher) independently extracted the data for each included study, discussed results, and resolved disagreements through discussions between investigators.

As noted in the prequal paper (20), extracted items were (1) process evaluation research design type, (2) process evaluation type, (3) process evaluation outcomes, (4) overall methods of data collection, (5) qualitative methods of data collection, (6) quantitative methods of data collection, (7) thematic analysis of process evaluation terms, (8) theories, frameworks, or conceptual models used, and (9) timing of data collection. In addition to these items extraction, elements and criteria of the REAIM and PRISM were extracted such as Reach, Effectiveness, Adoption, Implementation, Maintenance, Internal & External Contextual Factors.

For a study to be considered to meet reach, the study needed to possesses any of the following sub-constructs such as describing the target population; demographic, behavioral information about the target population; method to identify the target population; recruitment strategies; inclusion & exclusion criteria; eligible, invited (exposed to recruitment); sample size, individual participation rate or participation/ attendance rate (sample size/ eligible invited potential participants); comparisons between the target population about the study sample; statistical comparisons between the target population and the study sample; cost of recruitment; and qualitative methods to measure reach.

To meet effectiveness, the study needed to have any 1 of criteria: percent attrition at follow up, qualitative methods to measure efficacy/effectiveness; clinical outcomes [i.e., hemoglobin A1C, blood sugar, BMI, weight, blood pressure, lipids, triglycerides, fats, cholesterol, healthcare utilization (ER hospitalization, routine checkups, healthcare costs and etc.)]; knowledge; self-efficacy; behavioral outcomes [i.e., Summary of Diabetes Self-Care Activities & changes in self-management behaviors (AADE 7 Self Care Behaviors)], and psychosocial indicators [i.e., PHQ, wellbeing, stress, mental health, quality of life, depression, social support, perception of illness, resilience, coping strategies, proactive coping, diabetes empowerment, perceived stress, depressive symptoms, Chronic Illness Resources Survey, illness perception questionnaire (IPQ), Diabetes Illness Representation Questionnaire (DIRQ), WHO Quality of Life, diabetes distress, and etc.], attitudes, beliefs, goal attainment; and health literacy.

For adoption, the study needed to have any 1 of the following criteria at either the setting or provider/staff level. Setting level criteria included eligible, invited potential settings number of participating settings; setting participation rate; description of the targeted location; inclusion/exclusion criteria of the setting description of the intervention location; method to identify setting; comparison between the target and participating settings; statistical comparisons between the targeted and participating settings, and the average # of persons served per setting. Provider/Staff Level criteria include eligible, invited potential providers (staff); number of participating providers (staff); provider (staff) participation rate, methods to identify target providers, level of expertise of providers; inclusion/exclusion criteria of the providers (staff); comparison between targeted and participating providers (staff); statistical comparisons between targeted and participating providers (staff); measures of cost adoption; dissemination beyond originally planned; and qualitative measures to measure adoption.

For implementation, the study needed to meet any 1 of the following criteria: theory-based; engagement to inform intervention; # of intervention contacts; timing of intervention contacts; duration of intervention contacts; extent protocol delivered as intended (fidelity); consistency of implementation across settings or providers; tailoring/adaptations of the intervention; participant/attendance completion rates; measure of intervention costs; and qualitative method to measure implementation.

For maintenance, the study needed to meet either individual or organizational level criteria. Individual level criteria include outcome measures at some duration after intervention termination; attrition/loss to follow up of individuals; and qualitative methods to measure individual maintenance of the intervention. Organizational level criteria are alignment with organization mission; intervention alignment with the organization’s mission; institutionalization of program after completion of study; maintenance of the program after completion of the study; attrition/loss follow up of settings; and qualitative methods to measure organizational maintenance/sustainability.

Internal and External Contextual Factors were examined. Internal contextual factors include recipient and intervention characteristics. Recipient characteristics include patient characteristics (demographics disease burden, competing demands, knowledge & beliefs) and organizational characteristics (organizational health and culture; management support and communication; shared goals and cooperation; clinical leadership, systems and training; date and decision support; staffing and incentives; and expectation of sustainability). Tables 1–25 provide information about REAIM & PRISM items extracted from each individual study.

**Table 1:** REACH table #1.

RE-AIM element	Criteria	Keyserling et al. (2002) (36)	Melkus et al. (2004) (55)	Rosal et al. (2005) (38)	Wang and Chan (2005) (50)	Mauldon et al. (2006) (66)	Stetson et al. (2006) (49)	Feathers et al. (2007) (83)	Thoolen et al. (2007, 2008) (91, 92)	Vincent et al. (2007) (39)	Klug et al. (2008) (67)	Schillinger et al. (2008) (44)	Utz et al. (2008) (53)	Samuel-Hodge et al. (2009) (37)
REACH	Described target population	X	X		X		X	X		X			X	
	Demographic, behavioral information about target population	X	X	X	X	X	X	X	X	X	X	X	X	X
	Method to identify the target population									X		X		
	Recruitment strategies	X	X	X		X		X		X		X	X	X
	Inclusion criteria	X	X	X	X	X		X	X	X	X			X
	Exclusion criteria		X	X	X				X	X		X		X
	Eligible, invited (exposed to recruitment) potential participants									X		X		
	Sample size	X	X					X		X		X		
	Individual participation rate OR participation/attendance rate (sample size/ eligible invited potential participants)	X	X	X	X	X		X	X	X	X	X		X
	Comparisons between the target population and the study sample													
	Statistical comparisons between the target population and the study sample													
	Cost of recruitment		X											
	Qualitative methods to measure reach										X			

**Table 2:** REACH table #2.

RE-AIM element	Criteria	Steinhardt et al. (2009) (69)	Comellas et al. (2010) (68)	Dontje and Forrest (2011) (56)	Greenhalgh et al. (2011) (41)	Silva et al. (2011) (71)	Smith et al. (2011) (89) and Paul et al. (2013) (90)	Choi and Rush (2012) (57)	Reitz et al. (2012) (59)	Rygg et al. (2012) (88) and Rise et al. (2013) (87)	Sun et al. (2012) (12)	Botes et al. (2013) (97), Serfontein and Mash (2013) (99), and Mash et al. (2014) (98)	Islam et al. (2013) (75)	Sinclair et al. (2013) (102, 103)
REACH	Described target population			X		X	X				X	X		
	Demographic, behavioral information about target population	X			X	X		X	X	X	X	X	X	X
	Method to identify the target population													
	Recruitment strategies		X	X	X	X		X		X		X	X	X
	Inclusion criteria	X	X	X	X	X	X	X	X	X	X	X	X	X
	Exclusion criteria	X		X	X		X			X			X	
	Eligible, invited (exposed to recruitment) potential participants													
	Sample size		X				X			X				
	Individual participation rate OR participation/attendance rate (sample size/eligible invited potential participants)	X		X	X	X		X	X	X	X	X		X
	Comparisons between the target population and the study sample													
	Statistical comparisons between the target population and the study sample													
	Cost of recruitment													
	Qualitative methods to measure reach						X							

**Table 3:** REACH table #3.

RE-AIM element	Criteria	Tang et al. (2013, 2015) (95, 96)	Van der Does and Mash (2013) (72)	Miller et al. (2014) (86)	Swavely et al. (2014) (77)	Zheng et al. (2014) (74)	Gamboa Moreno et al. (2016) (30)	Lee et al. (2016) (33)	Vissenberg et al. (2016, 2017) (93, 94)	Akhter et al. (2017) (76)	Brunk et al. (2017) (29)	Odgers-Jewell et al. (2017) (100, 101)
REACH	Described target population		X					X	X			
	Demographic, behavioral information about target population	X	X	X	X	X	X	X	X	X		X
	Method to identify the target population											
	Recruitment strategies	X		X			X	X	X	X	X	X
	Inclusion criteria	X		X	X		X	X	X	X	X	X
	Exclusion criteria						X		X			
	Eligible, invited (exposed to recruitment) potential participants											
	Sample size				X			X				X
	Individual participation rate OR participation/attendance rate (sample size/eligible invited potential participants)	X				X		X	X			
	Comparisons between the target population and the study sample											
	Statistical comparisons between the target population and the study sample											
	Cost of recruitment											
	Qualitative methods to measure reach											

**Table 4:** REACH table #4.

RE-AIM element	Criteria	Pacheco et al. (2017) (73)	Schoenberg et al. (2017) (42)	Whitehead et al. (2017) (32)	Aziz et al. (2018) (58)	Taggart et al. (2018) (43)	Gucciardi et al. (2019) (82)	Han et al. (2019) (54)	Liu et al. (2019) (78)	Witry et al. (2019) (40)	McElfish et al. (2020) (70)
REACH	Described target population					X	X				
	Demographic, behavioral information about target population	X	X				X	X	X		X
	Method to identify the target population										
	Recruitment strategies		X		X	X	X	X	X	X	
	Inclusion criteria	X	X	X	X	X		X	X	X	X
	Exclusion criteria	X	X	X				X	X		X
	Eligible, invited (exposed to recruitment) potential participants		X		X	X				X	
	Sample size				X					X	
	Individual participation rate OR participation/attendance rate (sample size/ eligible invited potential participants)		X					X	X	X	X
	Comparisons between the target population and the study sample										
	Statistical comparisons between the target population and the study sample										
	Cost of recruitment										
	Qualitative methods to measure reach										

**Table 5:** REACH table #5.

RE-AIM element	Criteria	Andersen, 2021 (51)	Brady et al. (2021) (65)	Brennan et al. (2021, 2022) (31, 104)	Chen et al. (2021) (45)	Goff et al. (2021) (63)	Musial et al. (2021) (61)	Naik et al. (2022) (85)	Shiyanbola et al. (2022) (79)	Biber (2023) (81)	Binesh et al. (2023) (62)
REACH	Described target population										
	Demographic, behavioral information about target population	X	X	X	X	X	X	X	X	X	X
	Method to identify the target population										
	Recruitment strategies	X	X	X		X	X		X		X
	Inclusion criteria	X	X	X	X	X	X		X		X
	Exclusion criteria		X	X	X	X	X		X		X
	Eligible, invited (exposed to recruitment) potential participants			X							
	Sample size	X		X				X	X		
	Individual participation rate OR participation/attendance rate (sample size/eligible invited potential participants)	X	X	X	X	X	X	X	X		X
	Comparisons between the target population and the study sample										
	Statistical comparisons between the target population and the study sample										
	Cost of recruitment										
	Qualitative methods to measure reach										

**Table 6:** REACH table #6.

RE-AIM element	Criteria	Carillo, 2023	Changsieng et al. (2023) (47)	Eshete, 2023 (60)	Nederveld et al. (2023) (35)	Ngan et al. (2023) (48)	Reagan et al. (2023) (80)	Shrodes et al. (2023) (84)	Winkley et al. (2023) (64)	Zhang et al. (2023) (46)
REACH	Described target population	X								
	Demographic, behavioral information about target population	X	X	X			X	X		
	Method to identify the target population									
	Recruitment strategies		X			X			X	X
	Inclusion criteria		X				X			
	Exclusion criteria		X							
	Eligible, invited (exposed to recruitment) potential participants									
	Sample size			X						
	Individual participation rate OR participation/attendance rate (sample size/eligible invited potential participants)		X		X	X	X	X	X	X
	Comparisons between the target population and the study sample									
	Statistical comparisons between the target population and the study sample									
	Cost of recruitment									
	Qualitative methods to measure reach				X				X	

**Table 7:** EFFECTIVENESS table #1.

RE-AIM element	Criteria	Keyserling et al. (2002) (36)	Melkus et al. (2004) (55)	Wikblad et al. (2004) (27)	Rosal et al. (2005) (38)	Wang and Chan (2005) (50)	Mauldon et al. (2006) (66)	Stetson et al. (2006) (49)	Feathers et al. (2007) (83)	Thoolen et al. (2007, 2008) (91, 92)
EFFECTIVENESS	Percent attrition at follow-up									
	Qualitative methods to measure efficacy/effectiveness	X (dose received)	X (dose received)	X (dose received)	X (dose received)	X (dose received)	X (dose received)	X (dose received)	X (dose received)	X (dose received)
	Clinical outcomes [i.e., HBA1C, blood sugar, BMI, weight, blood pressure, lipids, triglycerides, fats, cholesterol, healthcare utilization (ER, hospitalization, routine check-ups, healthcare costs and etc.)]		X		X	X	X			
	Knowledge		X				X			
	Self-efficacy		X					X		X
	Behavioral [i.e., summary of diabetes self-care activities & changes in self-management behaviors (AADE 7 self care behaviors)]				X			X		
	Quality of life indicators [i.e., PHQ, wellbeing, stress, mental health, quality of life, depression, social support, perception of illness, resilience, coping strategies, proactive coping, diabetes empowerment, perceived stress, depressive symptoms, chronic illness resources survey, illness perception questionnaire (IPQ), diabetes illness representation questionnaire (DIRQ), WHO quality of life, diabetes distress, and etc.]		X		X	X	X	X		X
	Attitudes & beliefs						X (health belief & language acculturation)			X
	Health literacy									

**Table 8:** EFFECTIVENESS table #2.

RE-AIM element	Criteria	Vincent et al. (2007) (39)	Klug et al. (2008) (67)	Schillinger et al. (2008) (44)	Utz et al. (2008) (53)	Samuel-Hodge et al. (2009) (37)	Steinhardt et al. (2009) (69)	Comellas et al. (2010) (68)	Dontje and Forrest (2011) (56)	Greenhalgh et al. (2011) (41)
EFFECTIVENESS	Percent attrition at follow-up									
	Qualitative methods to measure efficacy/effectiveness	X	X (dose received)		X (dose received)	X (dose received)	X (dose received)		X (dose received)	
	Clinical outcomes [i.e., HBA1C, blood sugar, BMI, weight, blood pressure, lipids, triglycerides, fats, cholesterol, healthcare utilization (ER, hospitalization, routine check-ups, healthcare costs and etc.)]	X		X	X	X	X		X	X
	Knowledge	X						X		
	Self-Efficacy	X	X		X					X
	Behavioral [i.e., summary of diabetes self-care activities & changes in self-management behaviors (AADE 7 self care behaviors)]	X	X	X	X	X	X	X	X	
	Quality of life indicators [i.e., PHQ, wellbeing, stress, mental health, quality of life, depression, social support, perception of illness, resilience, coping strategies, proactive coping, diabetes empowerment, perceived stress, depressive symptoms, chronic illness resources survey, illness perception questionnaire (IPQ), diabetes illness representation questionnaire (DIRQ), WHO quality of life, diabetes distress, and etc.]			X			X	X		X
	Attitudes & beliefs		X (self-rated health)		X (goal attainment)					
	Health literacy									

**Table 9:** EFFECTIVENESS table #3.

RE-AIM element	Criteria	Silva et al. (2011) (71)	Smith et al. (2011) (89) and Paul et al. (2013) (90)	Choi and Rush (2012) (57)	Reitz et al. (2012) (59)	Rygg et al. (2012) (88) and Rise et al. (2013) (87)	Botes et al. (2013) (97), Serfontein and Mash (2013) (99), and Mash et al. (2014) (98)	Islam et al. (2013) (75)	Sinclair et al. (2013) (102, 103)	Tang et al. (2013, 2015) (95, 96)	Van der Does and Mash (2013) (72)	Miller et al. (2014) (86)
EFFECTIVENESS	Percent attrition at follow-up											
	Qualitative methods to measure efficacy/effectiveness	X (dose received)	X (dose received)	X (dose received)		X (dose received)	X (dose received)	X (dose received)	X (dose received)		X (dose received)	X (dose received)
	Clinical outcomes [i.e., HBA1C, blood sugar, BMI, weight, blood pressure, lipids, triglycerides, fats, cholesterol, healthcare utilization (ER, hospitalization, routine check-ups, healthcare costs and etc.)]	X	X	X	X	X	X		X	X		X
	Knowledge			X		X						
	Self-efficacy			X		X	X					X
	Behavioral [i.e., summary of diabetes self-care activities & changes in self-management behaviors (AADE 7 self care behaviors)]	X		X		X	X	X	X		X	X
	Quality of life indicators [i.e., PHQ, wellbeing, stress, mental health, quality of life, depression, social support, perception of illness, resilience, coping strategies, proactive coping, diabetes empowerment, perceived stress, depressive symptoms, chronic illness resources survey, illness perception questionnaire (IPQ), diabetes illness representation questionnaire (DIRQ), WHO quality of life, diabetes distress, and etc.]		X	X		X	X		X	X		
	Attitudes & beliefs	X										
	Health literacy											

**Table 10:** EFFECTIVENESS table #4.

RE-AIM element	Criteria	Swavely et al. (2014) (77)	Zheng et al. (2014) (74)	Gamboa Moreno et al. (2016) (30)	Lee et al. (2016) (33)	Vissenberg et al. (2016, 2017) (93, 94)	Whitehead et al. (2017) (32)	Akhter et al. (2017) (76)	Brunk et al. (2017) (29)	Odgers-Jewell et al. (2017) (100, 101)	Pacheco et al. (2017) (73)
EFFECTIVENESS	Percent attrition at follow-up										
	Qualitative methods to measure efficacy/effectiveness	X (dose received)	X (dose Received)	X (dose received)	X (dose received)	X (dose received)	X (dose received)	X (dose received)	X (dose received)	X (dose received)	X (dose received)
	Clinical outcomes [i.e., HBA1C, blood sugar, BMI, weight, blood pressure, lipids, triglycerides, fats, cholesterol, healthcare utilization (ER, hospitalization, routine check-ups, healthcare costs and etc.)]	X		X				X		X	X
	Knowledge	X						X	X	X	
	Self-efficacy	X		X				X		X	
	Behavioral [i.e., summary of diabetes self-care activities & changes in self-management behaviors (AADE 7 self care behaviors)]	X		X							
	Psychosocial Indicators (i.e., PHQ, wellbeing, stress, mental health, quality of life, depression, social support, perception of illness, resilience, coping strategies, proactive coping, diabetes empowerment, perceived stress, depressive symptoms, chronic illness resources survey, illness perception questionnaire (IPQ), diabetes illness representation questionnaire (DIRQ), WHO quality of life, diabetes distress, and etc.)			X				X		X	
	Attitudes, beliefs, goal attainment and etc.										
	Health literacy										

**Table 11:** EFFECTIVENESS table #5.

RE-AIM element	Criteria	Whitehead et al. (2017) (32)	Schoenberg et al. (2017) (42)	Aziz et al. (2018) (58)	Taggart et al. (2018) (43)	Gould et al. (2019) (28)	Gucciardi et al. (2019) (82)	Han et al. (2019) (54)	Liu et al. (2019) (78)	Witry et al. (2019) (40)	McElfish et al. (2020) (70)
EFFECTIVENESS	Percent attrition at follow-up										
	Qualitative methods to measure efficacy/effectiveness	X (dose received)	X (dose received)		X (dose received)	X (dose received)	X (dose received)		X (dose received)	X (dose received)	
	Clinical outcomes [i.e., HBA1C, blood sugar, BMI, weight, blood pressure, lipids, triglycerides, fats, cholesterol, healthcare utilization (ER, hospitalization, routine check-ups, healthcare costs and etc.)]		X	X	X			X	X		X
	Knowledge		X					X	X		
	Self-efficacy		X	X				X	X		
	Behavioral [i.e., summary of diabetes self-care activities & changes in self-management behaviors (AADE 7 self care behaviors)]		X	X				X	X	X	
	Psychosocial indicators [i.e., PHQ, wellbeing, stress, mental health, quality of life, depression, social support, perception of illness, resilience, coping strategies, proactive coping, diabetes empowerment, perceived stress, depressive symptoms, chronic illness resources survey, illness perception questionnaire (IPQ), diabetes illness representation questionnaire (DIRQ), WHO quality of life, diabetes distress, problem areas in diabetes (PAID) and etc.]		X	X	X			X			
	Attitudes, beliefs, goal attainment and etc.				X (perceptions about DM)					X	
	Health literacy							X			

**Table 12:** EFFECTIVENESS table #6.

RE-AIM element	Criteria	Andersen, 2021 (60)	Brady et al. (2021) (65)	Brennan et al. (2021, 2022) (31, 104)	Chen et al. (2021) (45)	Goff et al. (2021) (63)	Musial et al. (2021) (61)	Naik et al. (2022) (85)	Shiyanbola et al. (2022) (79)	Biber (2023) (81)	Carillo, 2023
EFFECTIVENESS	Percent attrition at follow-up										
	Qualitative methods to measure efficacy/effectiveness		X (dose received)	X (dose received)		X (dose received)	X (dose received)		X (dose received)		X (dose received)
	Clinical outcomes [i.e., HBA1C, blood sugar, BMI, weight, blood pressure, lipids, triglycerides, fats, cholesterol, healthcare utilization (ER, hospitalization, routine Check-ups, healthcare costs and etc.)]	X	X		X	X			X	X	X
	Knowledge					X		X			
	Self-efficacy		X	X	X	X			X		X
	Behavioral [i.e., summary of diabetes self-care activities & changes in self-management behaviors (AADE 7 Self Care Behaviors)]	X		X	X	X		X	X	X	X
	Psychosocial indicators [i.e., PHQ, wellbeing, stress, mental health, quality of life, depression, social support, peer support, perception of illness, resilience, coping strategies, proactive coping, diabetes empowerment, perceived stress, depressive symptoms, chronic illness resources survey, illness perception questionnaire (IPQ), diabetes illness representation questionnaire (DIRQ), WHO quality of life, diabetes distress, problem areas in diabetes (PAID) and etc.]		X	X		X		X	X	X	
	Attitudes, beliefs, goal attainment, achievement and etc.							X	X (DM Health Belief & Provider Communication)		
	Health literacy								X		

**Table 13:** EFFECTIVENESS table #7.

RE-AIM element	Criteria	Binesh et al. (2023) (62)	Changsieng et al. (2023) (47)	Eshete, 2023 (60)	Ngan et al. (2023) (48)	Reagan et al. (2023) (80)	Shrodes et al. (2023) (84)	Winkley et al. (2023) (64)	Zhang et al. (2023) (46)
EFFECTIVENESS	Percent attrition at follow-up								
	Qualitative methods to measure efficacy/effectiveness	X (dose received)	X (dose received)		X (dose received)	X (dose received)	X (dose received)	X (dose received)	
	Clinical outcomes [i.e., HBA1C, blood sugar, BMI, weight, blood pressure, lipids, triglycerides, fats, cholesterol, healthcare utilization (ER, hospitalization, routine check-ups, healthcare costs and etc.)]		X	X				X	X
	Knowledge		X			X			
	Self-efficacy		X		X				
	Behavioral [i.e., summary of diabetes self-care activities & changes in self-management behaviors (AADE 7 self care behaviors)]		X	X	X				X
	Psychosocial Indicators [i.e., PHQ, wellbeing, stress, mental health, quality of life, depression, social support, peer support, perception of illness, resilience, coping strategies, proactive coping, diabetes empowerment, perceived stress, depressive symptoms, chronic illness resources survey, illness perception questionnaire (IPQ), diabetes illness representation questionnaire (DIRQ), WHO Quality of Life, diabetes distress, problem areas in diabetes (PAID) and etc.]				X	X		X	X
	Attitudes, beliefs, goal attainment, achievement and etc.								
	Health literacy					X			

**Table 14:** ADOPTION table #1.

RE-AIM element	Criteria	Utz et al. (2008) (53)	Smith et al. (2011) (89) and Paul et al. (2013) (90)	Tang et al. (2013, 2015) (95, 96)	Whitehead et al. (2017) (32)	Aziz et al. (2018) (58)
ADOPTION (setting level)	Eligible, invited potential settings					X
	Number of participating settings					X
	Setting participation rate					X
	Description of the targeted location	X				
	Inclusion/exclusion criteria of the setting					
	Description of Intervention Location					
	Method to identify the setting					
	Comparisons between the target and participating settings					
	Statistical comparisons between the targeted and participating settings					
	Average # of persons served per setting					
ADOPTION (provider/staff level)	Eligible, invited potential providers (staff)				X	
	Number of participating providers (staff)	X		X		
	Provider (staff) participation rate			X		
	Method to identify target providers			X		
	Level of expertise of providers					
	Inclusion/exclusion criteria of the providers (staff)		X	X		
	Comparison between targeted and participating providers (staff)					
	Statistical comparisons between targeted and participating providers (staff)					
	Measures of cost adoption					
	Dissemination beyond originally planned					
	Qualitative measures to measure adoption		X			

**Table 15:** IMPLEMENTATION table #1.

RE-AIM element	Criteria	Melkus et al. (2004) (55)	Mauldon et al. (2006) (66)	Feathers et al. (2007) (83)	Utz et al. (2008) (53)	Steinhardt et al. (2009) (69)	Greenhalgh et al. (2011) (41)	Silva et al. (2011) (71)	Smith et al. (2011) (89) and Paul et al. (2013) (90)	Botes et al. (2013) (97), Serfontein and Mash (2013) (99), and Mash et al. (2014) (98)	Sinclair et al. (2013) (102, 103)
IMPLEMENTATION	Theory-based			X		X					
	Engagement to inform intervention			X							
	# of Intervention contacts				X	X			X	X	
	Timing of intervention contacts					X					
	Duration of intervention contacts				X	X					
	Extent protocol delivered as intended (fidelity)	X	X	X			X		X	X	X
	Consistency of Implementation across settings or providers							X			
	Tailoring/adaptions of the Intervention			X	X	X					
	Participant/attendance Completion rates										
	Measure of Intervention costs				X						
	Qualitative method to measure implementation							X			

**Table 16:** IMPLEMENTATION Table #2.

RE-AIM element	Criteria	Swavely et al. (2014) (77)	Akhter et al. (2017) (76)	Brunk et al. (2017) (29)	Aziz et al. (2018) (58)	Taggart et al. (2018) (43)	Gucciardi et al. (2019) (82)	Han et al. (2019) (54)	McElfish et al. (2020) (70)	Brennan et al. (2021, 2022) (31, 104)	Goff et al. (2021) (63)	Shiyanbola et al. (2022) (79)
IMPLEMENTATION	Theory-based		X					X		X		
	Engagement to inform intervention		X						X			
	# of Intervention Contacts (% of perfect intervention delivery completed)						X	X	X			
	Timing of intervention contacts			X								
	Duration of intervention contacts	X	X	X				X	X			
	Extent protocol delivered as intended (fidelity)				X	X	X			X	X	
	Consistency of implementation across settings or providers											
	Tailoring/adaptations of the intervention	X		X				X	X			
	Participant/attendance completion rates											
	Measure of intervention costs							X				
	Qualitative method to measure implementation					X		X				X

**Table 17:** IMPLEMENTATION table #3.

RE-AIM element	Criteria	Shiyanbola et al. (2022) (79)	Nederveld et al. (2023) (35)	Shrodes et al. (2023) (84)
IMPLEMENTATION	Theory-based			
	Engagement to inform intervention			
	# of Intervention contacts (% of perfect intervention delivery completed)			
	Timing of intervention contacts			
	Duration of intervention contacts			
	Extent protocol delivered as intended (fidelity)			
	Consistency of implementation across settings or providers			
	Tailoring/adaptations of the intervention		X	X
	Participant/attendance completion rates			
	Measure of intervention costs			
	Qualitative method to measure implementation	X	X	

**Table 18:** MAINTENANCE table #1.

RE-AIM element	Criteria	Melkus et al. (2004) (55)	Rosal et al. (2005) (38)	Wang and Chan (2005) (50)	Mauldon et al. (2006) (66)	Klug et al. (2008) (67)	Steinhardt et al. (2009) (69)	Comellas et al. (2010) (68)	Greenhalgh et al. (2011) (41)	Silva et al. (2011) (71)	Choi and Rush (2012) (57)	Rygg et al. (2012) (88) and Rise et al. (2013) (87)	Botes et al. (2013) (97), Serfontein and Mash (2013) (99), and Mash et al. (2014) (98)
MAINTENANCE (individual)	Follow up outcome measures at some duration after intervention termination	X	X	X	X	X	X	X	X	X	X	X	X
	Attrition/loss to follow up of individuals						X						
	Qualitative methods to measure individual maintenance of the intervention									X		X	
MAINTENANCE (organizational)	Alignment with organization mission												
	Intervention alignment with the organization’s mission												
	Institutionalization of program after completion of study												
	Maintenance of the program after completion of the study												
	Attrition/loss to follow up of settings Qualitative methods to measure organizational maintenance/sustainability												

**Table 19:** MAINTENANCE table #2.

*RE-AIM element*	Criteria	Sinclair et al. (2013) (102, 103)	Miller et al. (2014) (86)	Gamboa Moreno et al. (2016) (30)	Pacheco et al. (2017) (73)	Schoenberg et al. (2017) (42)	Whitehead et al. (2017) (32)	Aziz et al. (2018) (58)	Liu et al. (2019) (78)	Brennan et al. (2021, 2022) (31, 104)	Chen et al. (2021) (45)	Goff et al. (2021) (63)	Changsieng et al. (2023) (47)
MAINTENANCE (individual)	Follow up outcome measures at some duration after intervention termination		X	X	X	X		X	X	X	X	X	X
	Attrition/loss to follow up of individuals												
	Qualitative methods to measure individual maintenance of the intervention						X						
MAINTENANCE (organizational)	Alignment with organization mission												
	Intervention alignment with the organization’s mission												
	Institutionalization of program after completion of study												
	Maintenance of the program after completion of the study	X											
	Attrition/loss to follow up of settings Qualitative methods to measure organizational maintenance/ sustainability												

**Table 20:** MAINTENANCE table #3.

RE-AIM element	Criteria	Winkley et al. (2023) (64)	Zhang et al. (2023) (46)
MAINTENANCE (individual)	Follow up outcome measures at some duration after intervention termination	X	X
	Attrition/Loss to follow up of individuals		
	Qualitative methods to measure individual maintenance of the intervention		
MAINTENANCE (organizational)	Alignment with organization mission		
	Intervention alignment with the organization’s mission		
	Institutionalization of program after completion of study		
	Maintenance of the program after completion of the study		
	Attrition/ Loss to follow up of settings Qualitative methods to measure organizational maintenance/ sustainability		

**Table 21:** INTERNAL CONTEXT: recipient characteristics table #1.

RE-AIM element	Criteria	Wikblad et al. (2004) (27)	Rosal et al. (2005) (38)	Klug et al. (2008) (67)	Steinhardt et al. (2009) (69)	Comellas et al. (2010) (68)	Greenhalgh et al. (2011) (41)	Silva et al. (2011) (71)	Smith et al. (2011) (89) and Paul et al. (2013) (90)	Botes et al. (2013) (97), Serfontein and Mash (2013) (99), and Mash et al. (2014) (98)	Islam et al. (2013) (75)	Van der Does and Mash (2013) (72)	Gamboa Moreno et al. (2016) (30)	Aziz et al. (2018) (58)
INTERNAL CONTEXT: patient characteristics	Demographics		X	X		X		X						X
	Disease burden			X	X									
	Competing demands			X				X						
	Knowledge & beliefs	X												
INTERNAL CONTEXT: organizational characteristics	Organizational health and culture						X	X				X		
	Management support and communication						X	X		X		X	X	
	Shared goals and cooperation						X	X	X					
	Clinical leadership													
	Systems and training					X		X		X	X			
	Date and decision support													
	Staffing and incentives					X		X				X		
	Expectation of sustainability											X		

**Table 22:** INTERNAL CONTEXT: recipient characteristics table #2.

RE-AIM element	Criteria	McElfish et al. (2020) (70)	Carillo, 2023	Nederveld et al. (2023) (35)
INTERNAL CONTEXT: patient characteristics	Demographics		X	
	Disease burden			
	Competing demands			
	Knowledge & beliefs			
INTERNAL CONTEXT: organizational characteristics	Organizational health and culture			
	Management support and communication			
	Shared goals and cooperation	X		X
	Clinical leadership			X
	Systems and training			X
	Date and decision support			
	Staffing and incentives	X		X
	Expectation of sustainability			

**Table 23:** INTERNAL CONTEXT: intervention characteristics table #1.

RE-AIM element	Criteria	Klug et al. (2008) (67)	Steinhardt et al. (2009) (69)	Comellas et al. (2010) (68)	Silva et al. (2011) (71)	Islam et al. (2013) (75)	Van der Does and Mash (2013) (72)	Aziz et al. (2018) (58)	Gould et al. (2019) (28)	McElfish et al. (2020) (70)	Brennan et al. (2021, 2022) (31, 104)
INTERNAL CONTEXT: patient perspectives (values)	Patient centeredness	X		X	X	X	X			X	
	Provides patient choices	X					X				
	Addresses patient barriers		X		X	X			X	X	X
	Seamlessness of transition between program elements										
	Service and access								X		
	Burden (complexity and cost)										
	Feedback of results										
INTERNAL CONTEXT: organizational perspectives (values)	Readiness			X				X			
	Strength of the evidence base										
	Addresses barriers of frontline staff										
	Coordination across departments and specialties										
	Burden (complexity and cost)	X									
	Usability and adaptability										
	Trialability and reversibility										
	Ability to observe results			X	X						

**Table 24:** EXTERNAL CONTEXT: table #1.

RE-AIM element	Criteria	Utz et al. (2008) (53)	Schoenberg et al. (2017) (42)	Carillo, 2023	Shrodes et al. (2023) (84)
EXTERNAL CONTEXT	Policy				
	Resources				X
	Guidelines				
	Incentives	X	X	X	

**Table 25:** Stratification of REAIM & PRISM evaluation outcomes (k = 68; *n* = 78).

Process evaluation outcome**	Number of studies (k)	Number of articles (*n*)
Reach	66	76
Effectiveness*	66	76
Adoption	5	7
Implementation	24	30
Maintenance	25	31
Internal contextual factor	19	23
External contextual factor	4	4

*Includes studies that did not aim to assess the outcome but provided results for and vice versa. **Multiple studies included multiple process evaluation outcomes.

The Mixed Method Appraisal Tool (MMAT) was used to critically appraise each included study’s quality. This instrument evaluates five types of empirical studies: qualitative research, non-randomized studies, randomized controlled trials, quantitative descriptive studies, and mixed methods studies. It is intended for use in systematic mixed-method reviews (25, 26). Based on MMAT’s classification of empirical studies, the studies included in this review composed of more than one article were categorized as a combination of the research designs of their separate articles (i.e., 1 RCT + 1 QUAL-type study). The same tool and strategy were used in the prequel paper (20).

### 2.5 Data synthesis

Statistical pooling, or meta-analysis, was shown to be inappropriate because of the differences in study designs, different process assessments, and DSMPs outcome evaluations. To create a comprehensive summary of the included studies, a narrative synthesis of the studies was conducted for this evaluation utilizing the extracted elements.

## 3 Results

Sixty-eight studies (k) in 78 articles (*n*) (k = 68; *n* = 78) met our inclusion criteria (27–104). Throughout the results section, the letter “k” will refer to the number of studies while “n” will refer to the number of articles. Each component was analyzed based on reported criteria to assess their impact on intervention uptake and sustainability.

### 3.1 Study characteristics

The RE-AIM and PRISM frameworks were used to assess the 68 studies that satisfied the inclusion requirements (27–104). These studies assessed various aspects of diabetes self-management programs, including Reach (k = 66; *n* = 76), Effectiveness (k = 66; *n* = 76), Adoption (k = 5; *n* = 7), Implementation (k = 24; *n* = 30), Maintenance (k = 25; *n* = 31), Internal Contextual Factors (k = 19; *n* = 23), and External Contextual Factors (k = 4; *n* = 4). Tables 1–26 provide information about these results.

**Table 26:** REAIM & PRISM overview (k = 68; *n* = 78).

Author (year)	Reach	Effectiveness	Adoption	Implementation	Maintenance	Internal contextual factor	External contextual factor	Total # of elements used
Keyserling et al. (2002) (36)	X	X (dose received)						**2**
Melkus et al. (2004) (55)	X	X		X	X			**4**
Wikblad et al. (2004) (27)		X (dose received)				X		**2**
Rosal et al. (2005) (38)	X	X			X	X		**4**
Wang and Chan (2005) (50)	X	X			X			**3**
Mauldon et al. (2006) (66)	X	X		X	X			**4**
Stetson et al. (2006) (49)	X	X						**2**
Feathers et al. (2007) (83)	X	X		X				**3**
Thoolen et al. (2007, 2008) (91, 92)	X	X						**2**
Vincent et al. (2007) (39)	X	X						**2**
Klug et al. (2008) (67)	X	X			X	X		**4**
Schillinger et al. (2008) (44)	X	X						**2**
Utz et al. (2008) (53)	X	X	X	X			X	**5**
Samuel-Hodge et al. (2009) (37)	X	X						**2**
Steinhardt et al. (2009) (69)	X	X		X	X			**4**
Comellas et al. (2010) (68)	X	X			X	X		**4**
Dontje and Forrest (2011) (56)	X	X						**2**
Greenhalgh et al. (2011) (41)	X	X		X	X	X		**5**
Silva et al. (2011) (71)	X	X			X	X		**4**
Smith et al. (2011) (89) and Paul et al. (2013) (90)	X	X	X	X		X		**5**
Choi and Rush (2012) (57)	X	X			X			**3**
Reitz et al. (2012) (59)	X	X						**2**
Rygg et al. (2012) (88) & Rise et al. (2013) (87)	X	X			X			**3**
Sun et al. (2012) (12)	X							**1**
Botes et al. (2013) (97), Serfontein and Mash (2013) (99), and Mash et al. (2014) (98)	X	X		X		X		**4**
Islam et al. (2013) (75)	X	X				X		**3**
Sinclair et al. (2013) (102, 103)	X	X		X	X			**4**
Tang et al. (2013, 2015) (95, 96)	X	X	X					**3**
Van der Does and Mash (2013) (72)	X	X				X		**3**
Miller et al. (2014) (86)	X	X			X			**3**
Swavely et al. (2014) (77)	X	X		X				**3**
Zheng et al. (2014) (74)	X	X						**2**
Gamboa Moreno et al. (2016) (30)	X	X			X	X		**4**
Lee et al. (2016) (33)	X	X (dose received)						**2**
Vissenberg et al. (2016, 2017) (93, 94)	X	X						**2**
Akhter et al. (2017) (76)	X	X		X				**3**
Brunk et al. (2017) (29)	X	X		X				**3**
Odgers-Jewell et al. (2017) (100, 101)	X	X						**2**
Pacheco et al. (2017) (73)	X	X			X			**3**
Schoenberg et al. (2017) (42)	X				X		X	**3**
Whitehead et al. (2017) (32)	X	X	X		X			**4**
Aziz et al. (2018) (58)	X	X	X	X	X	X		**6**
Taggart et al. (2018) (43)	X	X (dose received)		X				**3**
Gould et al. (2019) (28)		X (dose received)				X		**2**
Gucciardi et al. (2019) (82)	X	X		X				**3**
Han et al. (2019) (54)	X	X		X				**3**
Liu et al. (2019) (78)	X	X			X			**3**
Witry et al. (2019) (40)	X	X (dose received)						**2**
McElfish et al. (2020) (70)	X	X		X		X		**4**
Andersen 2021 (51)	X	X						**2**
Brady et al. (2021) (65)	X	X						**2**
Brennan et al. (2021, 2022) (31, 104)	X	X		X	X	X		**5**
Chen et al. (2021) (45)	X	X			X			**3**
Goff et al. (2021) (63)	X	X		X	X			**4**
Musial et al. (2021) (61)	X	X						**2**
Naik et al. (2022) (85)	X	X						**2**
Shiyanbola et al. (2022) (79)	X	X		X				**3**
Biber (2023) (81)	X	X						**2**
Binesh et al. (2023) (62)	X	X						**2**
Carillo, 2023	X	X				X	X	**4**
Changsieng et al. (2023) (47)	X	X			X			**3**
Eshete (2023) (60)	X	X						**2**
Nederveld et al. (2023) (35)	X			X		X		**3**
Ngan et al. (2023) (48)	X	X						**2**
Reagan et al. (2023) (80)	X	X						**2**
Shrodes et al. (2023) (84)	X	X		X				**3**
Winkley et al. (2023) (64)	X	X			X			**3**
Zhang et al. (2023) (46)	X	X			X			**3**

There was significant diversity in the number of RE-AIM and PRISM framework elements reported by each of the 68 studies that were part of this review. One study (k = 1) included only 1 outcome domain, while 24 studies (k = 24) reported 2 outcomes, and 24 studies (k = 24) addressed 3 outcomes. A total of 14 studies (k = 14) reported 4 outcomes, 4 studies (k = 4) reported 5 outcomes, and 1 study (k = 1) included 6 outcomes. These results show that there is a range of comprehensiveness in DSMP evaluation, with most of the research concentrating on a small subset of implementation dimensions.

### 3.2 Reach

Sixty-six studies (k = 66; *n* = 76) evaluated reach (29–104). Nineteen studies (k = 19; *n* = 25) met the criteria related to describing the target population (33, 34, 36, 39, 43, 49, 50, 52, 53, 55, 56, 71, 72, 82, 83, 89, 90, 93, 94, 97–99). Fifty-five studies (k = 55; *n* = 69) met the criteria related to demographic, behavioral information about target population (12, 30, 31, 33, 34, 36–39, 41, 42, 44, 45, 47, 49–54, 57, 59–63, 65–67, 69–77, 79–88, 91–104). Two studies (k = 2; *n* = 2) included methods to identify the target population (39, 44). Forty-four studies (k = 44; *n* = 52) included recruitment strategies (12, 29–31, 33, 36–44, 46–48, 51, 53–58, 61–66, 68, 71, 75, 76, 79, 82, 83, 86–88, 93–104). Fifty-one studies included information on inclusion criteria (k = 51; *n* = 61) (12, 29–33, 36–43, 45, 47, 50–52, 54–59, 61–63, 65–71, 73, 75–77, 79, 80, 83, 86–104) while twenty-nine studies included information on exclusion criteria (k = 29; *n* = 34) (30–32, 37–39, 41, 42, 44, 45, 47, 50, 54–56, 61–63, 65, 69, 70, 73, 75, 78, 79, 87–94, 104). Seven studies (k = 7; *n* = 8) included information on eligible, invited, potential participants (31, 39, 40, 42–44, 58, 104) while eighteen studies included information on sample size (k = 18; *n* = 22) (31, 33, 36, 39, 40, 44, 51, 55, 58, 60, 68, 77, 79, 83, 85, 87–90, 100, 101, 104). Forty-seven studies (k = 47; *n* = 55) included criteria related to individual participation rate or participation/attendance rate (31, 33, 35–42, 44–48, 50–52, 54–57, 59, 61–67, 69–71, 74, 78–80, 83–85, 87, 88, 91–99, 102–104). One study (k = 1; *n* = 1) (55) included criteria related to cost of recruitment and five studies included criteria related to qualitative methods to measure reach (k = 5; *n* = 7) (35, 64, 67, 89, 90, 93, 94). None of the studies included criteria related to comparisons between the target population and the study sample and statistical comparisons between the target population and study sample.

### 3.3 Effectiveness

Sixty-six studies in seventy-six articles (k = 66; *n* = 76) assessed effectiveness (27–34, 36–51, 53–104). None of the studies include criteria related to percent attrition at follow up. Fifty-three studies (k = 53; *n* = 61) include criteria related to qualitative methods to measure efficacy/ effectiveness (27–34, 36–40, 42, 43, 47–50, 53, 55–57, 61–67, 69, 71–80, 82–84, 86–94, 97–104). Forty-three studies (k = 43; *n* = 49) include clinical outcomes (30, 34, 37–39, 41–47, 50, 51, 53–60, 63–66, 69–71, 73, 76–79, 81, 86–90, 95–103) while seventeen studies (k = 17; *n* = 19) include knowledge-related outcomes (29, 39, 42, 47, 54, 55, 57, 63, 66, 68, 76–78, 80, 85, 87, 88, 100, 101). Twenty-seven studies (k = 27; *n* = 31) include self-efficacy outcomes (30, 34, 39, 41, 42, 45, 47–49, 53–55, 57, 58, 63, 65, 67, 76–79, 86–88, 91, 92, 97–101). Thirty-eight studies (k = 38; *n* = 42) include behavioral outcomes (12, 30, 31, 34, 37–40, 42, 44–49, 51, 53, 54, 56–58, 60, 63, 67–69, 71, 72, 75, 77, 79, 81, 85–88, 97–99, 102–104). Thirty-four studies (k = 34; *n* = 43) included criteria related to psychosocial indicators (30, 31, 34, 38, 41–44, 46, 48–50, 54, 55, 57, 58, 63–66, 68, 69, 76, 79–81, 85, 87–92, 95–104). Nine studies (k = 9; *n* = 10) included criteria related to attitudes, beliefs, goal attainment, and achievement (40, 43, 53, 66, 67, 71, 79, 85, 91, 92) while three studies (k = 3; *n* = 3) include criteria related to health literacy (54, 79, 80).

### 3.4 Adoption

Five studies included information about adoption (k = 5; *n* = 7) (32, 53, 58, 89, 90, 95, 96). Two studies included setting level adoption criteria (53, 58). Aziz included information about eligible, invited potential settings; number of participating settings; and setting participation rates (58). Utz included information about the description of the targeted location (53).

On the other hand, four studies included provider/staff level adoption criteria (32, 53, 89, 90, 95, 96). Whitehead included information on eligible, invited potential providers (staff) (32); Utz included information on number of participating providers (staff) (53). One study included information on number of participating providers (staff); provider (staff) participation rate; method to identify target providers; and inclusion/exclusion criteria of the providers (staff) (95, 96). Finally, one study included information on inclusion/exclusion criteria of providers (staff) and qualitative measures to measure adoption (89, 90).

### 3.5 Implementation

Twenty-four studies assessed implementation (k = 24; *n* = 30) (29, 31, 35, 41, 43, 53–55, 58, 63, 66, 69–71, 76, 77, 79, 82–84, 89, 90, 93, 94, 97–99, 102–104). Five studies (k = 5; *n* = 6) met the criteria theory-based (31, 54, 69, 76, 83, 104); three studies met the criteria related to engagement to inform the intervention (70, 76, 83) and seven studies met the criteria related to the number of intervention contacts (% of perfect intervention delivery completed) (k = 7; *n* = 10) (53, 54, 69, 70, 82, 89, 90, 97–99). Two studies met the criteria timing of intervention contacts (k = 2; *n* = 2) (29, 69); seven studies met the criteria duration of intervention contacts (k = 7; *n* = 7) (29, 53, 54, 69, 70, 76, 77); and twelve studies (k = 12; *n* = 17) met the criteria related to extent protocol delivered as intended (fidelity) (31, 41, 43, 55, 58, 63, 66, 82, 83, 89, 90, 97–99, 102–104). One study (k = 1; *n* = 1) included criteria on consistency of implementation across settings or providers (71); nine studies included criteria on tailoring/adaptations of the intervention (k = 9; *n* = 9) (29, 35, 53, 54, 69, 70, 77, 83, 84). None of the criteria included information on participant/attendance completion rates. Two (k = 2; *n* = 2) included information on measures of intervention cost (53, 54) and five (k = 5; *n* = 5) included information on qualitative methods to measure implementation (35, 43, 54, 71, 79).

### 3.6 Maintenance

Maintenance was evaluated by 25 studies in total, with an emphasis on individual and organizational maintenance criteria (k = 25; *n* = 31) (30–32, 38, 41, 42, 45–47, 50, 55, 57, 58, 63, 64, 66–69, 71, 73, 78, 86–88, 97–99, 102–104). Twenty-four studies (k = 24; *n* = 30) include information on follow up outcome measures at some duration after intervention termination (12, 30, 31, 38, 41, 42, 45–47, 50, 55, 57, 58, 63, 64, 66–69, 71, 73, 86–88, 97–99, 102–104). One study included information on attrition/loss to follow up of individuals (k = 1; *n* = 1) (69) and three studies included qualitative methods to measure individual maintenance of the intervention (k = 3; *n* = 4) (32, 71, 87, 88). On the other hand, one study included information related to organizational maintenance specifically maintenance of the program after completion of the study (k = 1; *n* = 2) (102, 103).

### 3.7 Contextual factors

#### 3.7.1 Internal contextual factors

##### 3.7.1.1 Recipient characteristics

Eight studies included information on patient characteristics (demographics, disease burden, competing demands, knowledge and beliefs) (27, 34, 38, 58, 67–69, 71). When stratified, six studies included information on demographics (34, 38, 58, 67, 68, 71), two studies on disease burden (67, 71), two on competing demands (67, 71), and one on knowledge & beliefs (27).

Ten studies included information on organizational characteristics (30, 35, 41, 68, 70–72, 75, 89, 90, 97–99). When stratified, three focused on organizational health and culture (41, 71, 72), five on management support and communication (30, 41, 71, 72, 97–99), five on shared goals and cooperation (35, 41, 70, 71, 89, 90), one on clinical leadership (35); five on systems and training (35, 68, 70, 71, 97–99), none on date and decision support; five on staffing and incentives (35, 68, 70–72); and one on expectation of sustainability (72).

##### 3.7.1.2 Intervention characteristics

Nine studies (k = 9; *n* = 10) included information on patient perspectives (values) (28, 31, 67–72, 75, 104). Six studies included information on patient centeredness (67, 68, 70–72, 75). Two studies included information on patient choices (67, 72). Six studies (k = 6; *n* = 7) included information related to addressing patient barriers (28, 31, 69–71, 75, 104). One study included information related to service and access (28). None of the studies included information about the seamlessness of transition between program elements, burden (complexity and cost), and feedback of results.

Three studies included information on organizational perspectives (values) (58, 67, 68). Two studies included information on organizational readiness (58, 68). One study included information on burden (complexity and cost) (67) and another study included information on ability to observe results (68).

#### 3.7.2 External contextual factors

One study included information on resources (84) while three studies included information on incentives (34, 42, 53).

### 3.8 Critical appraisal

The critical appraisal of included articles for this study is also listed in the prequel paper (20). Using the MMAT (Mixed Methods Appraisal Tool), numerous articles (*n* = 57) satisfied at least 60% of the MMAT quality standards (27–33, 35–37, 39, 41–56, 58–63, 71–73, 76, 78, 79, 82, 83, 86–99, 101, 104). Among these, Twenty-one articles (*n* = 21) satisfied 60% of the quality criteria (*n* = 21) (37, 38, 44, 46, 48, 67, 69, 73, 75, 79, 80, 84, 87, 88, 92, 94, 97, 100, 101, 104). Seventeen articles had 80% of their quality criteria met (*n* = 17) (36, 39, 41, 44, 47, 48, 50, 52, 53, 55, 56, 59, 61–63, 89, 95). Nineteen articles had 100% of their quality criteria met (*n* = 19) (27–30, 32, 33, 35, 37, 45, 46, 58, 60, 87, 90, 93, 94, 97, 99, 100). Conversely, some articles fell below 60% in quality, with 21 studies meeting 40% or less of the criteria (34, 38, 40, 57, 64–70, 74, 75, 77, 79–81, 84, 85, 102, 103). Of these, three article had 0% of their quality criteria met (*n* = 3) (66, 80, 81) while seven had 20% of their quality criteria met (*n* = 7) (34, 64, 65, 79, 84, 85, 103). Eleven articles had 40% of their quality criteria met (*n* = 11) (38, 40, 57, 67–70, 74, 75, 77, 102). Appendix C of the Supplementary File shows the tabulations of each study’s critical appraisal categorized by their respective study design.

## 4 Discussion

### 4.1 Summary of findings

This systematic review applied the RE-AIM and PRISM frameworks to 68 studies of conventional, group-based DSMPs, revealing that adoption, implementation, maintenance, and contextual reporting remain substantially underrepresented compared to reach and effectiveness. This focus on patient-level outcomes is in line with earlier DSMP research (2–5, 10), but it restricts the field’s capacity to evaluate long-term sustainability and scalability. The uneven reporting across RE-AIM/PRISM domains also suggests fragmented program evaluations, reducing opportunities for generalization across contexts.

By showing that DSMP research specifically leaves out provider-level adoption, external context, and cost data—all essential elements for converting programs into standard practice—our findings build on previous studies of process evaluations in chronic disease interventions (13–17, 78). This review’s methodical use of PRISM highlighted that organizational capability, policy background, participant variables, and intervention content are all critical to implementation success (18, 19).

### 4.2 Reach

Nearly all studies reported demographic and inclusion criteria, aligning with observations by Schinckus et al. (16) regarding descriptive reach data dominance in DSME research. However, there was little documentation of recruitment expenses, participation rates, and qualitative descriptions of barriers—all important measures of accessibility and equity. This exclusion is more noticeable than in assessments conducted in school-based vaccination and neurological rehabilitation settings (13, 14). Recent equity-focused DSMP implementations—such as the HED program among predominantly Black/African American participants (105), the Project Dulce model serving low-income, underserved communities (106), and a DSME intervention in a Federally Qualified Health Center for medically underserved patients (107)—highlight how equity-oriented design can improve reach and inclusivity.

### 4.3 Effectiveness

Consistent with earlier findings, most DSMPs in this review demonstrated improvements in HbA1C, self-efficacy, and behavior (2–5, 10). However, there was a significant lack of attrition reporting and long-term follow-up, which is contrary to the 2022 DSMES guidelines that prioritize extended outcome monitoring (10). For instance, a Chinese community-based intervention sustained elevated self-efficacy, glucose monitoring, and physical activity levels two years post-intervention—though there was some decline from immediate post-program peaks (108). Similarly, an empowerment-based intervention among African American participants not only maintained these behaviors one year after a two-year intervention but also showed further improvements in glycemic and lipid outcomes (109). Conversely, a DSMP delivered to underserved Latino patients yielded immediate A1C improvements that reverted to baseline after one year, despite sustained reductions in LDL and blood pressure (110). Although DSMPs can yield long-lasting benefits, program effects are not consistently maintained, as these contradictory results demonstrate. The absence of systematic long-term tracking limits understanding of outcome durability and may reflect resource constraints, publication priorities, or a prevailing focus on short-term efficacy in DSMP research.

### 4.4 Adoption

Only five studies assessed adoption, and few provided detailed information about provider- or setting-level characteristics. This finding aligns with Durlak and DuPre, who noted that adoption is commonly underreported in implementation research (11), but it also indicates an even greater deficit in DSMP literature compared with other public health interventions (13–15). Possible explanations include a historic emphasis on patient-level outcomes in DSME research and an implicit assumption that adoption occurs automatically once a program is delivered, rather than as a distinct process requiring evaluation. Notably, the Partners in Care program, which implemented a culturally tailored DSMES intervention for Native Hawaiians and Pacific Islanders, demonstrated that peer-educator-led models can be successfully integrated into community-based and low-resource clinical settings by systematically assessing adoption, feasibility, fidelity, acceptability, and sustainability (102).

### 4.5 Implementation

Implementation fidelity and program structure were moderately well documented, confirming Durlak and DuPre’s assertion that these elements are typically prioritized in implementation reporting (11). However, cost analyses, documentation of adaptation processes, and use of theory-driven frameworks were inconsistently addressed. This contrasts with more recent PRISM-guided evaluations in other chronic disease domains that emphasize explicit theoretical grounding and systematic tracking of adaptations (18). For example, the DSME-OK program—developed through the Intervention Mapping protocol and informed by the Information–Motivation–Behavioral Skills (IMB) model—demonstrated how a theory-driven approach can define behavioral objectives, guide intervention design, support implementation planning, and ensure rigorous evaluation (111).

### 4.6 Maintenance

Though individual-level maintenance was often assessed through post-intervention outcomes, organizational sustainability was rarely evaluated—echoing McCreight et al.’s (2019) concern regarding the institutionalization of public health programs (18). Compared with other chronic disease interventions, this gap appears even wider in DSMPs, likely due to reliance on time-limited grants and the lack of reimbursement structures. For example, Maryland’s global budget payment model supported the development of the Center for Clinical Resources (CCR), an integrated diabetes care initiative that provided interdisciplinary self-management support, addressed unmet social needs, achieved durable HbA₁c reductions, and decreased emergency department and inpatient utilization. This program illustrates how alternative payment and policy mechanisms can foster long-term sustainability beyond short-term funding cycles. In the absence of such structural integration, DSMP longevity remains uncertain.

### 4.7 Contextual factors

Internal contextual factors such as patient demographics and organizational culture were variably reported—echoing findings by Liu et al. (78). However, external influences—policy environment, resources, and incentives—were even less frequently documented, more so than in prior implementation reviews (13–15). This oversight matters greatly, as external context often dictates program adaptability in low-resource settings. The imbalance likely stems from DSMPs’ clinical orientation, which foregrounds individual-voice data over institutional or policy dynamics.

### 4.8 Strengths and limitations of the review

This review’s thorough evaluation of several implementation domains using accepted evaluation frameworks is one of its main strengths. Findings are more broadly applicable when studies from various groups and environments are included. Nevertheless, there are several limitations, such as inconsistent reporting, with many studies neglecting program expenses, provider participation, or recruitment tactics. Effective comparison of interventions and the development of solid conclusions about best practices are hampered by this lack of detail (15, 16). Initially, the researchers only included English-language published publications. As a result, articles in other languages that are important to the goal of this study might be overlooked. However, evidence suggests that post-synthesis findings are not significantly affected by the exclusion of publications from non-English journals (112). Second, it is possible that process aspects were gathered and examined but were not included in published papers because of journal word count restrictions because the data collection methods of the included process evaluations did not go beyond the data presented in studies.

### 4.9 Implications for research and practice

The review’s conclusions have several important clinical practice and public health ramifications for the execution of DSMPs as well as the more general objectives of managing and preventing chronic diseases, especially among older adults. This study contributes uniquely to the existing literature by applying the RE-AIM/PRISM framework to traditional, group-based DSMPs, enabling a multidimensional assessment of both implementation processes and contextual influence elements often underreported in past research (18, 19). Unlike prior evaluations that focus predominantly on program effectiveness, this review underscores the need for evaluating processes such as organizational readiness, provider engagement, and contextual barriers to long-term sustainability.

According to the findings, coordinated diabetes self-management education and support (DSMES) programs that tackle implementation and sustainability issues are crucial (10). Long-term improvements in patient involvement and health outcomes have been shown in programs that integrate community-based efforts with peer-led assistance (2–5, 10). In line with suggestions for policy-driven initiatives, DSMES integration into existing healthcare infrastructures may also increase program adoption and patient engagement (9).

Crucially, this assessment highlights the healthcare community’s wider inability to address the underlying causes of the diabetes epidemic. For decades, healthcare systems have often normalized obesity, reassured patients that pharmacologic treatments alone can manage diabetes, hypertension, and arthritis, and failed to confront pervasive misinformation—such as the myth that physical activity is potentially dangerous for older adults by causing untoward injuries (113). On the other hand, structured exercise is one of the best non-pharmacologic ways to improve insulin sensitivity, cardiovascular health, and glycemic control, and scientific research repeatedly shows that physical activity at any level or type typically results in positive health outcomes and healthy aging across a variety of chronic illnesses and conditions (114). DSMPs that explicitly address physical activity, nutritional literacy, and behavioral goal setting can equip patients with the tools to counter these damaging narratives.

While there has been substantial research about diabetes prevention and management, future studies are needed that examine how DSMPs for older adults can effectively change behavior in an overburdened healthcare environment. We recommend greater attention to understanding the sources of and strategies for changing conflicting public health messages. In addition, health care providers and public health researchers should collaborate on research to identify best practices for implementing DSMP programs across a wide range of populations and environments. Our knowledge of how to successfully scale and sustain DSMES programs across the life-course that promote genuine, long-lasting health effects will be strengthened by a greater focus on mixed-method evaluations that look at participant attitudes, environmental variables, and system-level policies.

## 5 Conclusion

This systematic review emphasizes the benefits of employing the PRISM framework to assess conventional, group-based Diabetes Self-Management Programs (DSMPs) from an implementation science perspective. While evidence of clinical, behavioral, and psychosocial benefits remains strong, significant gaps persist in the reporting of process-level indicators such as adoption, maintenance, and contextual influences. These underreported domains are essential for understanding the scalability and sustainability of DSMPs across diverse healthcare settings.

Findings also point to administrative hurdles, a shortage of licensed educators, and billing restrictions as obstacles to practical implementation, especially in low-resource clinics and federally designated health facilities. Simultaneously, the expanding use of telehealth and hybrid program delivery offers chances to expand access while posing fresh difficulties about participation and digital equity.

Future evaluations should include standardized assessments of environmental, organizational, and delivery elements in addition to outcome reporting to progress research and practice. To ensure reimbursement, workforce development, and flexible program forms, policymakers and executives in the healthcare industry should encourage the incorporation of DSMPs into conventional care models. In the end, achieving the full potential of DSMPs to enhance health outcomes and lessen the burden of diabetes at scale requires thorough and context-aware implementation techniques.

## Data Availability

The raw data supporting the conclusions of this article will be made available by the authors, without undue reservation.
