# Supplementary material for: The application of the RE-AIM and PRISM framework to process evaluations of diabetes self-management programs: a systematic review and secondary analysis of literature
Source: Front Public Health. 2025 Dec 12;13:1588457. doi: 10.3389/fpubh.2025.1588457 (PMC12740919; doi:10.3389/fpubh.2025.1588457)
Supplement: Supplementary file 1 [file Supplementary_file_1.docx]

**Appendix A:** Mixed Method Appraisal Tool (MMAT) Tables

**Table A.1:** Distribution of MMAT Scores for Qualitative Articles (n= 12)

| **MMAT Score Distribution** | **Number of Articles** |
| --- | --- |
| 0% | 0 |
| 20% | 0 |
| 40% | 0 |
| 60% | 0 |
| 80% | 0 |
| 100% | 12 |

**Table A.2:** Distribution of MMAT Scores for Quantitative Descriptive (n= 2)

| **MMAT Score Distribution** | **Number of Articles** |
| --- | --- |
| 0% | 0 |
| 20% | 0 |
| 40% | 0 |
| 60% | 0 |
| 80% | 2 |
| 100% | 0 |

**Table A.3:** Distribution of MMAT Scores for Quantitative Non-Randomized Studies (n= 15)

| **MMAT Score Distribution** | **Number of Articles** |
| --- | --- |
| 0% | 0 |
| 20% | 0 |
| 40% | 1 |
| 60% | 4 |
| 80% | 7 |
| 100% | 3 |

**Table A.4:** Distribution of MMAT Scores for Quantitative Randomized Controlled Trials (n= 20)

| **MMAT Score Distribution** | **Number of Articles** |
| --- | --- |
| 0% | 0 |
| 20% | 1 |
| 40% | 2 |
| 60% | 7 |
| 80% | 7 |
| 100% | 3 |

**Table A.5:** Distribution of MMAT Scores for Mixed Method Studies (n= 30)

| **MMAT Score Distribution** | **Number of Articles** |
| --- | --- |
| 0% | 2 |
| 20% | 6 |
| 40% | 3 |
| 60% | 5 |
| 80% | 10 |
| 100% | 4 |

**Table A.6:** Associated Quality of Qualitative Studies (n=12)

| **Author (Year)** | **Screening Questions** | | **Qualitative** | | | | |
| --- | --- | --- | --- | --- | --- | --- | --- |
|  | **Are there clear research questions?** | **Do the collected data allow to address the research questions?** | **Is the qualitative approach**  **appropriate to answer the research question?** | **Are the qualitative data collection methods adequate to address the research question?** | **Are the findings adequately derived from the data?** | **Is the interpretation of results sufficiently substantiated by data?** | **Is there coherence between qualitative data sources, collection, analysis, and interpretation?** |
| **Wikblad (2004)** | Yes | Yes | Yes | Yes | Yes | Yes | Yes |
| **Botes (2013)** | Yes | Yes | Yes | Yes | Yes | Yes | Yes |
| **Paul (2013)** | Yes | Yes | Yes | Yes | Yes | Yes | Yes |
| **Rise (2013)** | Yes | Yes | Yes | Yes | Yes | Yes | Yes |
| **Serfontein (2013)** | Yes | Yes | Yes | Yes | Yes | Yes | Yes |
| **Lee (2016)** | Yes | Yes | Yes | Yes | Yes | Yes | Yes |
| **Vissenberg (2016)** | Yes | Yes | Yes | Yes | Yes | Yes | Yes |
| **Whitehead (2016)** | Yes | Yes | Yes | Yes | Yes | Yes | Yes |
| **Brunk (2017)** | Yes | Yes | Yes | Yes | Yes | Yes | Yes |
| **Odgers-Jewell (2017)** | Yes | Yes | Yes | Yes | Yes | Yes | Yes |
| **Gould (2019)** | Yes | Yes | Yes | Yes | Yes | Yes | Yes |
| **Nederveld (2023)** | Yes | Yes | Yes | Yes | Yes | Yes | Yes |

**Table A.7:** Associated Quality of Quantitative Randomized Controlled Trial Studies (n= 19)

| **Author (Year)** | **Screening Questions** | | **Quantitative Randomized Control Trials** | | | | |
| --- | --- | --- | --- | --- | --- | --- | --- |
|  | **Are there clear research questions?** | **Do the collected data allow to address the research questions?** | **Is the randomization appropriately performed?** | **Are the groups comparable at baseline?** | **Are there complete outcome data?** | **Are the outcome assessors blinded to the intervention provided?** | **Did the participants adhere to the assigned intervention?** |
| **Keyserling (2002)** | Yes | Yes | Yes | Yes | Yes | Can’t Tell | Yes |
| **Rosal (2005)** | Yes | Yes | Can’t Tell | Yes | No | Can’t Tell | Yes |
| **Thoolen (2007)** | Yes | Yes | Yes | Yes | No | Can’t Tell | Yes |
| **Thoolen (2008)** | Yes | Yes | Yes | Yes | No | Can’t Tell | Yes |
| **Vincent (2007)** | Yes | Yes | Yes | Yes | Yes | Can’t Tell | Yes |
| **Schillinger (2008)** | Yes | Yes | Yes | Yes | Yes | No | Yes |
| **Samuel-Hodge (2009)** | Yes | Yes | Yes | Yes | Yes | Yes | Yes |
| **Greenhalgh (2011)** | Yes | Yes | Yes | Yes | Yes | Can’t Tell | Yes |
| **Rygg (2011)** | Yes | Yes | Yes | Yes | No | Can’t Tell | Yes |
| **Smith (2011)** | Yes | Yes | Yes | Yes | Yes | Can’t Tell | Yes |
| **Sinclair (2013)** | Yes | Yes | Yes | No | No | No | No |
| **Mash (2014)** | Yes | Yes | Yes | Yes | No | No | Yes |
| **Schoenberg (2017)** | Yes | Yes | Can’t Tell | Yes | Yes | Can’t Tell | Yes |
| **Taggart (2017)** | Yes | Yes | Yes | Yes | No | Can’t Tell | Yes |
| **Witry (2019)** | Yes | Yes | Can’t Tell | Yes | No | Can’t Tell | Yes |
| **Brennan (2021)** | Yes | Yes | Yes | Yes | No | No | Yes |
| **Chen (2021)** | Yes | Yes | Yes | Yes | Yes | Yes | Yes |
| **Zhang**  **(2022)** | Yes | Yes | Yes | Yes | Yes | Yes | Yes |
| **Changsieng, 2023** | Yes | Yes | No | Yes | Yes | Yes | Yes |
| **Ngan, 2023** | Yes | Yes | Yes | Yes | Yes | Can’t Tell | Yes |

**Table A.8:** Associated Quality of Quantitative Non-Randomized Studies (n= 15)

| **Author (Year)** | **Screening Questions** | | **Quantitative Non-Randomized Studies** | | | | |
| --- | --- | --- | --- | --- | --- | --- | --- |
|  | **Are there clear research questions?** | **Do the collected data allow to address the research questions?** | **Are the participants representative of the target population?** | **Are measurements appropriate regarding both the outcome and intervention (or exposure)?** | **Are there complete outcome data?** | **Are there confounders accounted for in the design and analysis?** | **During the study period, is the intervention administered (or exposure occurred) as intended?** |
| **Melkus (2004)** | Yes | Yes | Yes | Yes | Yes | No | Yes |
| **Wang (2005)** | Yes | Yes | Yes | Yes | Yes | No | Yes |
| **Stetson (2006)** | Yes | Yes | No | Yes | Yes | No | Yes |
| **Utz (2008)** | Yes | Yes | Yes | Can’t Tell | Yes | Yes | Yes |
| **Dontje (2011)** | Yes | Yes | Yes | Yes | Yes | No | Yes |
| **Choi (2012)** | Yes | Yes | Can’t Tell | Yes | No | No | Yes |
| **Reitz (2012)** | Yes | Yes | Can’t Tell | Yes | Yes | Yes | Yes |
| **Sun (2012)** | Yes | Yes | Yes | Yes | Yes | No | Yes |
| **Tang (2013)** | Yes | Yes | Yes | Yes | Yes | No | Yes |
| **Moreno (2016)** | Yes | Yes | Yes | Yes | Yes | Yes | Yes |
| **Aziz (2018)** | Yes | Yes | Yes | Yes | Yes | Yes | Yes |
| **Han (2019)** | Yes | Yes | Yes | Yes | No | No | Yes |
| **Andersen**  **(2020)** | Yes | Yes | Yes | Yes | No | No | Yes |
| **Shiyanbola, 2022** | Yes | Yes | Yes | Yes | No | No | Yes |
| **Eshete, 2023** | Yes | Yes | Yes | Yes | Yes | Yes | Yes |

**Table A.9:** Associated Quality of Quantitative Descriptive (n= 2)

| **Author (Year)** | **Screening Questions** | | **Quantitative Non-Randomized Studies** | | | | |
| --- | --- | --- | --- | --- | --- | --- | --- |
|  | **Are there clear research questions?** | **Do the collected data allow to address the research questions?** | **Is the sampling strategy relevant to address the research question?** | **Is the sample representative of the target population?** | **Are the measurements appropriate?** | **Is the risk of nonresponse bias low?** | **Is the statistical analysis appropriate to answer the research question?** |
| **Binesh (2020)** | Yes | Yes | Yes | Yes | Yes | Can’t Tell | Yes |
| **Musial**  **(2022)** | Yes | Yes | Yes | Yes | Yes | Can’t Tell | Yes |

**Table A.10:** Associated Quality of Mixed Method Studies: Appraisal of Qualitative Portion (Part 1) (n=30)

| **Author (Year)** | **Screening Questions** | | **Qualitative** | | | | |
| --- | --- | --- | --- | --- | --- | --- | --- |
|  | **Are there clear research questions?** | **Is the qualitative approach appropriate to answer the research question?** | **Is the qualitative approach appropriate to answer the research question?** | **Are the qualitative data collection methods adequate to address the research question?** | **Are the findings adequately derived from the data?** | **Is the interpretation of results sufficiently substantiated by data?** | **Is there coherence between qualitative data sources, collection, analysis, and interpretation?** |
| **Mauldon (2006)** | Yes | Yes | Yes | Can’t Tell | Can’t Tell | Can’t Tell | Can’t Tell |
| **Two Feathers (2007)** | Yes | Yes | Yes | Yes | Yes | No | Yes |
| **Klug (2008)** | Yes | Yes | Yes | Yes | Can’t Tell | Can’t Tell | Can’t Tell |
| **Steinhardt (2009)** | Yes | Yes | Yes | Yes | Yes | Yes | Can’t Tell |
| **Comellas (2010)** | Yes | Yes | Yes | Yes | Can’t Tell | Can’t Tell | Can’t Tell |
| **Silva (2011)** | Yes | Yes | Yes | Yes | Yes | Yes | Yes |
| **Islam (2013)** | Yes | Yes | Yes | Yes | Yes | No | Yes |
| **Tang (2014)** | Yes | Yes | Yes | Yes | Yes | No | Yes |
| **Pacheco (2017)** | Yes | Yes | Yes | Yes | Yes | Can’t Tell | Yes |
| **Swavely (2013)** | Yes | Yes | Yes | Yes | Yes | No | Yes |
| **Van der Does (2013)** | Yes | Yes | Yes | Yes | Yes | Yes | Yes |
| **Miller (2014)** | Yes | Yes | Yes | Yes | Yes | Yes | Can’t Tell |
| **Zheng (2014)** | Yes | Yes | Yes | Yes | Yes | Yes | Can’t Tell |
| **Vissenberg (2017)** | Yes | Yes | Yes | Yes | Yes | Yes | Yes |
| **Akhter (2017)** | Yes | Yes | Yes | Yes | Yes | Yes | Yes |
| **Odgers-Jewell (2017)** | Yes | Yes | Yes | Yes | Yes | Yes | Yes |
| **Gucciardi (2018)** | Yes | Yes | Yes | Yes | Yes | Yes | Yes |
| **Liu (2019)** | Yes | Yes | Yes | Yes | Yes | Yes | Yes |
| **McElfish (2020)** | Yes | Yes | Yes | Yes | Can’t Tell | Can’t Tell | Can’t Tell |
| **Sinclair (2020)** | Yes | Yes | Yes | Yes | Yes | Yes | Yes |
| **Brady (2021)** | Yes | Yes | Yes | Yes | Yes | Yes | Yes |
| **Brennan (2021)** | Yes | Yes | Yes | Yes | Yes | Yes | Yes |
| **Goff (2021)** | Yes | Yes | Yes | Yes | Yes | Yes | Yes |
| **Naik (2022)** | Yes | Yes | Yes | Yes | Yes | Yes | Yes |
| **Shiyanbola et. al, 2022b** | Yes | Yes | Yes | Yes | Yes | Yes | Yes |
| **Shrodes et. al, 2022** | Yes | Yes | Yes | Yes | Yes | Yes | Yes |
| **Biber et. al, 2023** | Yes | Yes | Unsure | Unsure | Unsure | Yes | Unsure |
| **Carillo et. al, 2023** | Yes | Yes | No | Yes | Yes | Yes | Yes |
| **Reagan et al. 2023** | Yes | Yes | No | No | No | No | No |
| **Winkley et al., 2023** | Yes | Yes | Yes | Yes | Unsure | Yes | Yes |

**Table A.11:** Associated Quality of Included Mixed Method Studies Studies: Appraisal of Quantitative Randomized Controlled Trial Studies (Part 2A) (n=3/30)

| **Author (Year)** | **Screening Questions** | | **Quantitative Randomized Control Trials** | | | | |
| --- | --- | --- | --- | --- | --- | --- | --- |
|  | **Are there clear research questions?** | **Do the collected data allow to address the research questions?** | **Is the randomization appropriately performed?** | **Are the groups comparable at baseline?** | **Are there complete outcome data?** | **Are the outcome assessors blinded to the intervention provided?** | **Did the participants adhere to the assigned intervention?** |
| **Brennan (2021)** | Yes | Yes | Yes | Yes | No | No | Yes |
| **Goff**  **(2021)** | Yes | Yes | Yes | Yes | Yes | No | Yes |
| **Winkley et al., 2023** | Yes | Yes | Yes | Yes | Yes | No | Yes |

**Table A.12:** Associated Quality of Mixed Method Studies Studies: Appraisal of Quantitative Non-Randomized Portion (Part 2B) (n=22/30)

| **Author (Year)** | **Screening Questions** | | **Quantitative Non-Randomized Studies** | | | | |
| --- | --- | --- | --- | --- | --- | --- | --- |
|  | **Are there clear research questions?** | **Are the participants representative of the target population?** | **Are the participants representative of the target population?** | **Are measurements appropriate regarding both the outcome and intervention (or exposure)?** | **Are there complete outcome data?** | **Are there confounders accounted for in the design and analysis?** | **During the study period, is the intervention administered (or exposure occurred) as intended?** |
| **Mauldon (2006)** | Yes | Yes | Yes | Yes | Yes | No | Yes |
| **Klug (2008)** | Yes | Yes | Yes | Yes | No | No | Yes |
| **Steinhardt (2009)** | Yes | Yes | No | Yes | No | No | Yes |
| **Comellas (2010)** | Yes | Yes | Yes | Yes | Yes | No | Yes |
| **Silva (2011)** | Yes | Yes | Yes | Yes | No | No | Yes |
| **Islam (2013)** | Yes | Yes | Yes | Yes | No | No | Yes |
| **Pacheco (2017)** | Yes | Yes | Yes | Yes | Can’t Tell | No | Yes |
| **Swavely (2013)** | Yes | Yes | Can’t Tell | Yes | No | No | Yes |
| **Van der Does (2013)** | Yes | Yes | No | Yes | Yes | No | Yes |
| **Miller (2014)** | Yes | Yes | Yes | Yes | Can’t Tell | No | Yes |
| **Tang (2014)** | Yes | Yes | Yes | Yes | No | No | Yes |
| **Zheng (2014)** | Yes | Yes | Yes | Yes | Yes | No | Yes |
| **Akhter (2017)** | Yes | Yes | Yes | Yes | No | No | Yes |
| **Odgers-Jewell (2017)** | Yes | Yes | Yes | Yes | No | No | Yes |
| **Liu (2019)** | Yes | Yes | Yes | Yes | Yes | No | Yes |
| **McElfish (2020)** | Yes | Yes | Yes | Yes | Yes | No | No |
| **Sinclair (2020)** | Yes | Yes | Yes | Yes | No | Can’t Tell | Yes |
| **Brady (2021)** | Yes | Yes | Yes | Yes | Yes | No | Yes |
| **Shiyanbola et. al, 2022b** | Yes | Yes | Yes | Yes | Yes | No | Yes |
| **Biber et. al, 2023** | Yes | Yes | Yes | Yes | Yes | Yes | Yes |
| **Carillo et. al, 2023** | Yes | Yes | Yes | Yes | Yes | No | Yes |
| **Reagan et al. 2023** | Yes | Yes | Yes | Yes | Yes | Yes | Yes |

**Table A.13:** Associated Quality of Mixed Method Studies Studies: Appraisal of Quantitative Descriptive Portion (Part 2C) (n=5/30)

| **Author (Year)** | **Screening Questions** | | **Quantitative Non-Randomized Studies** | | | | |
| --- | --- | --- | --- | --- | --- | --- | --- |
|  | **Are there clear research questions?** | **Are the participants representative of the target population?** | **Is the sampling strategy relevant to address the research question?** | **Is the sample representative of the target population?** | **Are the measurements appropriate?** | **Is the risk of nonresponse bias low?** | **Is the statistical analysis appropriate to answer the research question?** |
| **Two Feathers (2007)** | Yes | Yes | Yes | No | Yes | Yes | Yes |
| **Vissenberg (2017)** | Yes | Yes | Yes | Yes | Yes | Yes | Yes |
| **Gucciardi (2018)** | Yes | Yes | Yes | Yes | Yes | Yes | Yes |
| **Naik (2022)** | Yes | Yes | Yes | Yes | Yes | Unsure | Yes |
| **Shrodes (2022)** | Yes | Yes | Yes | Yes | Yes | Unsure | Yes |

**Table A.14:** Associated Quality of Mixed Method Studies (Part 3) (n=30)

| **Author (Year)** | **Screening Questions** | | **Mixed Method Studies** | | | | |
| --- | --- | --- | --- | --- | --- | --- | --- |
|  | **Are there clear research questions?** | **Do the collected data allow to address the research questions?** | **Is there an adequate rationale for using a mixed method design to address the research question?** | **Are the different components of the study effectively integrated to answer the research question?** | **Are the outputs of the integration of qualitative and quantitative components adequately interpreted?** | **Are divergences and inconsistencies between quantitative and qualitative results adequately addressed?** | **Do the different components of the study adhere to the quality criteria of each tradition of the methods involved?** |
| **Mauldon (2006)** | Yes | Yes | No | No | No | No | No |
| **Two Feathers (2007)** | Yes | Yes | No | Can’t Tell | Yes | Yes | Yes |
| **Klug (2008)** | Yes | Yes | Can’t Tell | Yes | Yes | Yes | Yes |
| **Steinhardt (2009)** | Yes | Yes | No | Yes | Yes | Yes | Can’t Tell |
| **Comellas (2010)** | Yes | Yes | No | Yes | Yes | Yes | Yes |
| **Silva (2011)** | Yes | Yes | No | Yes | Yes | Yes | Yes |
| **Islam (2013)** | Yes | Yes | No | Can’t Tell | Yes | Yes | Can’t Tell |
| **Odgers-Jewell (2017)** | Yes | Yes | No | Can’t Tell | Yes | Yes | Yes |
| **Pacheco (2017)** | Yes | Yes | Can’t Tell | Yes | Yes | No | Yes |
| **Swavely (2013)** | Yes | Yes | No | Yes | Yes | Yes | Yes |
| **Van der Does (2013)** | Yes | Yes | No | Yes | Yes | Yes | Yes |
| **Miller (2014)** | Yes | Yes | No | Yes | Yes | Yes | Yes |
| **Tang (2014)** | Yes | Yes | Can’t Tell | Yes | Yes | Yes | Yes |
| **Zheng (2014)** | Yes | Yes | No | Yes | No | Yes | No |
| **Vissenberg (2017)** | Yes | Yes | Yes | Yes | Yes | Yes | Yes |
| **Akhter (2017)** | Yes | Yes | Can’t Tell | Yes | Yes | Yes | Yes |
| **Gucciardi (2018)** | Yes | Yes | No | Can’t Tell | Yes | Yes | Yes |
| **Liu (2019)** | Yes | Yes | Yes | Yes | Yes | Yes | Yes |
| **McElfish (2020)** | Yes | Yes | Can’t Tell | Yes | Yes | Yes | Yes |
| **Sinclair (2020)** | Yes | Yes | No | Can’t Tell | Yes | No | Yes |
| **Brady (2021)** | Yes | Yes | No | No | No | Can’t Tell | Yes |
| **Brennan (2021)** | Yes | Yes | Yes | Yes | No | Yes | Yes |
| **Goff (2021)** | Yes | Yes | Yes | Yes | Yes | Yes | Yes |
| **Naik (2022)** | Yes | Yes | No | No | No | Unsure | Yes |
| **Shiyanbola et. al, 2022b** | Yes | Yes | No | No | No | No | Yes |
| **Shrodes (2022)** | Yes | Yes | No | No | No | No | Yes |
| **Biber et. al, 2023** | Yes | Yes | Yes | Yes | Yes | Yes | Yes |
| **Carillo et. al, 2023** | Yes | Yes | No | No | No | No | Yes |
| **Reagan et al., 2023** | Yes | Yes | No | No | No | No | No |
| **Winkley et al., 2023** | Yes | Yes | No | No | No | No | Yes |
