# Supplementary material for: The application of the RE-AIM and PRISM framework to process evaluations of diabetes self-management programs: a systematic review and secondary analysis of literature
Source: Front Public Health. 2025 Dec 12;13:1588457. doi: 10.3389/fpubh.2025.1588457 (PMC12740919; doi:10.3389/fpubh.2025.1588457)
Supplement: Supplementary file 2 [file Supplementary_file_2.docx]

**Appendix B: Search Strategies**

**Table B.1:** Search Strategy #1: Medline Ovid, Updated 01/12/2024

| 1 | exp Diabetes Mellitus/ |
| --- | --- |
| 2 | diabet*.ti,ab. |
| 3 | or/1-2 |
| 4 | exp Self Care/ or exp Self-Management/ |
| 5 | (self adj1 (care or manage*)).ti,ab. |
| 6 | selfcare.ti,ab. |
| 7 | (lifestyle* adj2 diab*).ti,ab. |
| 8 | or/4-7 |
| 9 | exp "Process Assessment (Health Care)"/ |
| 10 | ((implement* or process) adj1 (assess* or eval*)).ti,ab. |
| 11 | (implement* or acceptab* or feasibil*).ti,ab. |
| 12 | or/9-11 |
| 13 | 3 and 8 and 12 |

**Table B.2:** Search Strategy #2: Embase Ovid, Updated 01/12/2024

| 1 | exp Diabetes Mellitus/ |
| --- | --- |
| 2 | diabet*.ti,ab. |
| 3 | or/1-2 |
| 4 | exp self care/ |
| 5 | selfcare*.ti,ab. |
| 6 | (self adj1 manag*).ti,ab. |
| 7 | (lifestyle* adj2 diab*).ti,ab. |
| 8 | or/4-7 |
| 9 | (process adj1 (assess* or eval*)).ti,ab. |
| 10 | (implement* adj1 (assess* or eval*)).ti,ab. |
| 11 | or/9-10 |
| 12 | 3 and 8 and 11 |
| 13 | (implement* or acceptab* or feasibil*) |
| 14 | 3 and 8 and (11 or 13) |

**Table B.3:** Search Strategy #3: CINAHL (Ebsco), Updated 01/12/2024

| 1 | (MH "Diabetes Mellitus+") OR ( TI diabet* or AB diabet* ) |
| --- | --- |
| 2 | AND |
| 3 | (MH "Self-Management") or AB ((self n1 (care or manage*)) or selfcare* or (lifestyle* n2 diab*)) or TI ((self n1 (care or manage*)) or selfcare* or (lifestyle* n2 diab*)) |
| 4 | AND |
| 5 | (MH "Process Assessment (Health Care)+") OR ( TI ((process or implement*) n1 (assess* or evaluat*)) ) OR ( AB ((process or implement*) n1 (assess* or evaluat*)) ) OR AB ((implement* or acceptab* or feasibil*) or TI (implement* or acceptab* or feasibil*) |

**Table B.4:** Search Strategy #4: Academic Search, Updated 01/12/2024

| 1 | ( TI diabet* or AB diabet* ) |
| --- | --- |
| 2 | AB ((self n1 (care or manage*)) or selfcare* or (lifestyle* n2 diab*)) or TI ((self n1 (care or manage*)) or selfcare* or (lifestyle* n2 diab*)) |
| 3 | ( TI ((process or implement*) n1 (assess* or evaluat*)) ) OR ( AB ((process or implement*) n1 (assess* or evaluat*)) ) OR AB ((implement* or acceptab* or feasibil*) or TI (implement* or acceptab* or feasibil*) |

**Table B.5:** Search Strategy #5: PsycINFO, Updated 01/12/2024

| 1 | DE "Self-Management" OR AB ((self n1 (care or manage*)) or selfcare* or (lifestyle* n2 diab*)) or TI ((self n1 (care or manage*)) or selfcare* or (lifestyle* n2 diab*)) |
| --- | --- |
| 2 | AND |
| 3 | DE "Diabetes" OR DE "Diabetes Mellitus" OR DE "Type 2 Diabetes" OR ( TI diabet* or AB diabet* ) |
| 4 | AND |
| 5 | (TI ((process or implement*) n1 (assess* or evaluat*)) ) OR ( AB ((process or implement*) n1 (assess* or evaluat*)) )  OR AB ((implement* or acceptab* or feasibil*) or TI (implement* or acceptab* or feasibil*) |
