# Supplementary material for: The application of the RE-AIM and PRISM framework to process evaluations of diabetes self-management programs: a systematic review and secondary analysis of literature
Source: Front Public Health. 2025 Dec 12;13:1588457. doi: 10.3389/fpubh.2025.1588457 (PMC12740919; doi:10.3389/fpubh.2025.1588457)
Supplement: Supplementary file 3 [file Supplementary_file_3.docx]

**Figure 1:**

**PRISMA 2020 flow diagram for new systematic reviews which included searches of databases and other sources only**

Records identified from*:

Databases (n =10,229)

Other Sources (n = 7)

Records removed *before screening*:

Duplicate records removed (n =4,943)

Records screened.

(n = 5,293)

Records excluded.

(n = 4,884)

Reports sought for retrieval.

(n = 409)

Reports not retrieved.

(n =1)

Reports assessed for eligibility.

(n = 408)

Reports excluded (n=330):

1. Reports that are not a quantitative, qualitative, or mixed-method study (n =133)
2. Reports that are not a Process Evaluation (PE) or does not include at least 1 PE-related outcome. (n = 122)
3. Reports where DSME, DSMS or DSMES programs are not delivered in a traditional, face to face, group-based format (n = 35)
4. Reports not written in English (n=20)
5. Reports that do not focus on a DSME, DSMS, or DSMES intervention (n=13)
6. Reports that do not include participants at least 18 years with either T1DM OR T2DM (n=7)

Studies included in review.

(k=68) in (n=78) articles

**Identification**

**Screening**

**Included**

**Identification of studies via databases and other sources**

*From:*  Page MJ, McKenzie JE, Bossuyt PM, Boutron I, Hoffmann TC, Mulrow CD, et al. The PRISMA 2020 statement: an updated guideline for reporting systematic reviews. BMJ 2021;372:n71. doi: 10.1136/bmj.n71

For more information, visit: <http://www.prisma-statement.org/>
